# Verbascoside: An Efficient and Safe Natural Antibacterial Adjuvant for Preventing Bacterial Contamination of Fresh Meat

**DOI:** 10.3390/molecules27154943

**Published:** 2022-08-03

**Authors:** Chunyang Shi, Yangmin Ma, Lei Tian, Jingyi Li, Guaiping Qiao, Chang Liu, Wenqiang Cao, Chengyuan Liang

**Affiliations:** 1 School of Food and Bioengineering, Shaanxi University of Science & Technology, Xi’an 710021, China; shichunyang@sust.edu.cn (C.S.); lijingyimmc@aliyun.com (J.L.); guaipingqiao@aliyun.com (G.Q.); 2 A Shaanxi Research Institute of Agricultural Products Processing Technology, Xi’an 710021, China; 3 College of Chemistry & Chemical Engineering, Shaanxi University of Science & Technology, Xi’an 710021, China; mayangmin@sust.edu.cn; 4 College of Bioresources Chemical and Materials Engineering, Shaanxi University of Science & Technology, Xi’an 710021, China; leitian115@gmail.com; 5 Zhuhai Jinan Selenium Source Nanotechnology Co., Ltd., Hengqin New Area, Zhuhai 519000, China; sesource_cwq@163.com

**Keywords:** multidrug-resistant bacteria, antibiotics, adjuvant, multiple antibacterial mechanisms, safety evaluation, fresh meat, shelf life

## Abstract

Inappropriate and disproportionate antibiotic use contributes immensely to the development of antibiotic resistance in bacterial species associated with food contamination. Therefore, alternative strategies to treat multidrug-resistant (MDR) bacterial infections are urgently needed. In this study, verbascoside was shown to exhibit excellent antibacterial activity and synergistic effects in combination with cell wall synthesis-inhibiting antibiotics, indicating that it can be used as an adjuvant to restore or increase the activity of antibiotics against resistant pathogens. In a mechanistic study, higher concentrations of verbascoside resulted in a longer lag phase and a lower specific exponential-phase growth rate of bacteria. Furthermore, verbascoside exerted its antimicrobial activity through multiple mechanisms, including cell membrane dysfunction, biofilm eradication and changes in cell morphology. The promising antibacterial activity and in vitro safety assessment results suggested that verbascoside can be used as a food additive for fresh meat preservation. Treatment with medium and high doses of verbascoside caused significant bacterial death in meat samples, slowed the spoilage rate, and extended the shelf life. Collectively, verbascoside is expected to be useful as an antibiotic adjuvant to prevent or treat resistant bacteria-related infections and an alternative novel antimicrobial additive in the food industry.

## 1. Introduction

The contamination and deterioration of fresh food caused by pathogens and microorganisms in the process of refrigeration and sales is a major public health concern. For instance, in the fresh meat production and processing chain, the moist and nutrient-rich surface of fresh meat provides a favourable environment for microbial growth [1]. Fresh meat may carry foodborne pathogens, which can cause sickness and food safety problems. In addition, the drug resistance rate of foodborne pathogen contaminants has increased in recent years [2]. Resistant strains have been widely detected in the food processing industry and may compromise food safety and shelf life [3]. The rapid global rise in multidrug-resistant (MDR) bacteria is largely attributable to antibiotic overuse and misuse in the healthcare, veterinary and agricultural sectors [4]. A primary repercussion for individual health is that MDR microbes may cause diseases in the host, such as urinary tract infections [5,6,7]. Among all foodborne pathogenic bacteria, MDR *Staphylococcus aureus* (*S. aureus*) and *Pseudomonas aeruginosa* (*P. aeruginosa*) are notorious for their ability to cause a spectrum of acute food poisoning events and to contaminate food-processing equipment [8,9]. For a long time, the use of organic chemical additives as antibacterial agents has been a common practice in food preservation. However, the safety of chemical synthetic additives has been widely questioned in recent years. Accordingly, there is a pressing need to search for effective natural antimicrobial agents to replace synthetic preservatives or use them in combination to reduce the dosage of chemical reagents [10], thereby significantly alleviating the harm.

**Figure 1:**
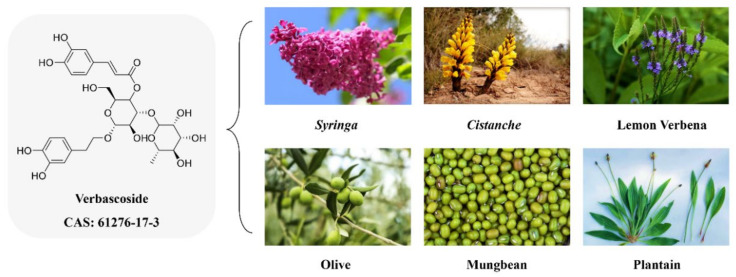
Chemical structure and natural sources of verbascoside.

Verbascoside is a caffeoyl phenylethanoid glycoside that has been found in 79 edible plants, including *Syringa* spp., lemon verbena, *Cistanche* spp., olive, mung bean, and plantain (Figure 1) [11]. Verbascoside has various physiological effects, such as anti-inflammatory, antioxidant and regulatory effects on cell apoptosis [12]. In addition, some studies have shown that verbascoside possesses antibacterial and antifungal activity; for example, plant extracts containing verbascoside have been shown to enhance the antibacterial effect of gentamicin against *S. aureus* and *Escherichia coli* (*E. coli*) [13]. Verbascoside combined with amphotericin B exhibits potent inhibitory activity against multiple fungal pathogens, including *Cryptococcus neoformans*, *Aspergillus* spp. and *Candida* spp. [14]. Nevertheless, the mechanism of the antibacterial action of verbascoside is not fully understood [15]. Therefore, verbascoside is mainly used in human healthcare to promote the proliferation of neural stem cells, but its application as an antibacterial agent in food has rarely been reported.

The aims of this study were to explore the antibacterial potential of verbascoside and investigate its safety and mechanisms as an antibacterial adjuvant for treating antibiotic-resistant bacteria. In addition, fresh meat is highly perishable, and therefore, requires optimized processing and storage systems. This study expands the application range of verbascoside in the field for reducing meat spoilage and provides a theoretical basis and practical application for a novel food additive with antimicrobial effects that can be applied in the food industry.

## 2. Materials and Methods

### 2.1. Reagents

Verbascoside (CAS: 61276-17-3) (purity ≥ 98%) was purchased from Chengdu Alfa Biotechnology Co., Ltd. (Chengdu, China). All other chemicals employed were of analytical or HPLC grade and used as supplied. Tryptone soya broth (TSB) and tryptone soya agar (TSA) were purchased from Guangdong Huankai Microbiology Technology Co., Ltd. (Guangzhou, China).

### 2.2. Bacterial Strains and Culture Conditions

*S. aureus* and *P. aeruginosa* are common pathogenic bacteria in foods and drinks. The standard strains *S. aureus* ATCC 25923 and *P. aeruginosa* ATCC 15442 were purchased from the American Type Culture Collection (ATCC, Manassas, VA, USA). The MDR *S. aureus* (SA-575 and SA-596) and MDR *P. aeruginosa* (PA-69 and PA-261) strains used were multiantibiotic-resistant isolates from patients with food poisoning. All bacteria were maintained at −80 °C in TSB containing 20% glycerol (*v*/*v*) [16]. Stock cultures were streaked on TSA and grown at 37 °C for 18 h prior to each experiment. One loopful of each bacterial culture was inoculated into TSB growth medium (30 mL) and incubated overnight at 37 °C.

### 2.3. MIC Determination

The MICs of verbascoside against all the experimental bacterial strains were measured by using the microplate dilution method [17]. The strains were grown aerobically at 37 °C in TSB until the optical density at 600 nm reached 0.5 (approximately 10^8^ CFU/mL). A high-concentration solution of verbascoside was prepared using TSB under sterile conditions. The stock solution was diluted to achieve the final concentration using a gradient dilution method. Then, a suspension of each bacterial strain (10 μL) was aliquoted into a well of a flat-bottom, clear 96-well plate (Corning, NY, USA) containing 190 μL of sample fluid at one of various concentrations, followed by incubation at 37 °C. Moxifloxacin, levofloxacin and ceftazidime were used as positive controls, while 10 µL of bacterial culture and 190 µL of TSB were used as negative controls. The control group wells were prepared using the same method used for the wells of the verbascoside groups. The final concentrations of verbascoside were 2500, 1250, 625, 312.5, 156.25, 78, 39, and 19.5 μg/mL in a 200 μL system, whereas the concentrations of antibiotics were 512, 256, 128, 64, 32, 16, 8 and 4 μg/mL, respectively. Following incubation of the plate for 24 h at 37 °C, the turbidity of the bacterial cultures in the wells was examined visually. The MIC values were determined as the minimum concentration at which no visible bacterial growth could be seen.

### 2.4. Evaluation of Synergistic Effects

The in vitro synergistic and antagonistic effects of verbascoside in combination with antibiotics, including vancomycin and ceftazidime, were determined using the checkerboard dilution method [18]. The MIC value was recorded as the lowest concentration that completely inhibited bacterial growth, either alone or in combination. Briefly, clear 96-well plates (Corning, NY, USA) were prepared with increasing concentrations of antibiotics in the columns and verbascoside in the rows. A bacterial suspension was inoculated into each well at approximately 5 × 10^5^ CFU/mL. The microplates were incubated for 24 h at 37 °C to allow bacterial growth and were visually inspected. Synergistic interactions were evaluated by determining the fractional inhibitory concentration index (FICI), which was calculated as follows: FICI = (MIC of agent A, tested in combination/MIC of agent A, tested alone) + (MIC of agent B, tested in combination/MIC of agent B, tested alone) [19]. The results were interpreted according to the following scale: FICI ≤ 0.5, synergistic; 0.5 < FICI < 1, partially synergistic; FICI = 1, additive; 1> FICI ≤ 4, neutral; and FICI > 4, antagonistic.

### 2.5. Bacterial Growth Curve Analysis

According to the procedure reported by Zhang et al. [20] with a few modifications, bacterial growth curves were constructed to evaluate the antibacterial activity of verbascoside. Suspensions of strains SA-596 and PA-69 (approximately 10^8^ CFU/mL, 100 μL) were inoculated into sterile TSB (100 mL). Verbascoside was added to the culture medium at a concentration of 1/2 × MIC, MIC, or 2 × MIC. Sterile TSB without verbascoside was used as the control. Then, all the cultures were grown in a shaking incubator (180 rpm) for 24 h at 37 °C. Bacterial growth was continuously measured every 1–2 h by assaying the optical density at 600 nm with an enzyme marker (Thermo Fisher Scientific, Vantaa, Finland).

### 2.6. Membrane Permeability Assay

#### 2.6.1. Intracellular ATP Concentrations

Intracellular ATP levels were measured with an ATP determination kit (Beyotime, Shanghai, China) according to a previously described method [21]. Briefly, overnight cultures of MDR *S. aureus* SA-596 and MDR *P. aeruginosa* PA-69 were harvested by centrifugation (6000× *g*) for 10 min. After being washed in 0.1 mol/L sterile phosphate-buffered saline (PBS, pH 7.0), the pellets were harvested by centrifugation. The bacterial cells were then suspended at a concentration of 10^8^ CFU/mL in PBS containing verbascoside at one of the various concentrations (0, MIC, or 2 × MIC). The solution was incubated at 37 °C for 30 min. Next, 1 mL of each of the above solutions containing different concentrations of verbascoside was distributed into three 1.5 mL centrifuge tubes. Then, 100 μL of lysis buffer was added to the cells in each tube to achieve full cleavage. After centrifugation, the supernatant serum was stored on ice until the assay. ATP detection buffer was added to white, opaque 96-well plates (100 μL/well, Corning, NY, USA) for 10 min to eliminate background fluorescence. Finally, each supernatant (20 μL) was transferred into individual wells, and light emission was measured (excitation/emission: 510/580 nm) using a multimode reader (Synergy H1, BioTek, Winooski, VT, USA).

#### 2.6.2. Intracellular pH (pH_in_) Measurement

The intracellular pH (pH_in_) was evaluated based on the method established by Shi et al. with some modifications [21]. Cells of MDR *S. aureus* SA-596 and MDR *P. aeruginosa* PA-69 were prepared as described in Section 2.6.1 and washed three times with 50 mM HEPES buffer (containing 5 mM EDTA, pH 8.0). Carboxyfluorescein diacetate succinimidyl ester (cFDA-SE; Sigma, St. Louis, MO, USA) was added to 50 mM HEPES buffer (10 mL) to a final concentration of 3.0 mM and incubated at 37 °C for 20 min. After incubation, the harvested cells were washed once in PBS and dispersed in 10 mL of PBS. After incubation, glucose was added at a final concentration of 10 mM, and the cells were incubated for half an hour at 37 °C to remove the unbound dye. The fluorescently labelled bacterial cells were resuspended in 5 mL of PBS containing verbascoside at one of the various concentrations (0, MIC, or 2 × MIC). Subsequently, the bacterial suspensions (10^8^ CFU/mL) were incubated in the dark at 37 °C for half an hour and then seeded into black, opaque 96-well plates (Corning, NY, USA). The fluorescence intensity was measured with a multimode reader (Synergy H1, BioTek, Winooski, VT, USA) at an excitation wavelength of 490 nm and an emission wavelength of 520 nm. After subtraction of the background fluorescence, the pH_in_ values corresponding to the fluorescence ratio were obtained based on the ratio of the fluorescence intensity at 520 nm using excitation wavelengths of 440 and 490 nm via calibration curves of cFDA-SE-loaded cells with buffers at different pH_in_ values.

#### 2.6.3. Membrane Potential Assay

Cell membrane potentials were determined using the fluorescence dye bis-(1,3-dibutylbarbituric acid) trimethine oxonol (DiBAC4 (3); Sigma, St. Louis, USA) according to methods described elsewhere [22] with minor modifications. Cells of MDR *S. aureus* SA-596 and MDR *P. aeruginosa* PA-69 were prepared as described in Section 2.6.1. Each pellet was resuspended in PBS (10^8^ CFU/mL) with one of the various concentrations of verbascoside (0, MIC, or 2 × MIC). Then, the samples were incubated for 30 min at 30 °C. A bacterial suspension (200 μL) and the fluorescent probe DiBAC4 (3) (1 mM final concentration) were added to each well of a black, opaque 96-well plate (Corning, NY, USA). The mixtures were then incubated at 37 °C for 30 min in the dark. Cell suspensions were analysed using a fluorescence spectrophotometer (BioTek, Winooski, VT, USA) with excitation and emission wavelengths of 488 nm and 518 nm, respectively. Changes in the fluorescence intensity of DiBAC4 (3) were recorded to monitor the membrane potential. All the values were corrected for cell number and background fluorescence.

#### 2.6.4. Extracellular Conductivity

The changes in membrane permeability were assayed by determining the extracellular conductivity of the supernatants obtained from the bacterial suspension treated with verbascoside as described in detail previously [22]. Cells of SA-596 and PA-69 (10^8^ CFU/mL, 0.1 mL) were inoculated into sterile TSB (100 mL), and both bacterial suspensions were treated with verbascoside at MIC or 2 × MIC. Bacterial suspensions without verbascoside were considered controls. The cultures were incubated in a shaking incubator (180 rpm) for 12 h at 37 °C. Samples (2 mL) were taken every hour. Following centrifugation at 6000× *g* at 4 °C for 10 min, the supernatant was collected. Finally, the extracellular conductivity was recorded using a conductometer (Thermo Fisher Scientific, Tokyo, Japan).

### 2.7. Bacterial Membrane Integrity

Cell membrane integrity was assessed based on methods described by Shi et al. with slight modifications using the LIVE/DEAD BacLight Bacterial Viability Kit (Waltham, MA, USA) [23]. Overnight cultures of SA-596 and PA-69 cells were established, and the pellets were resuspended in 2 mL of 0.85% NaCl solution. The cells were exposed to verbascoside at a concentration of 0, MIC or 2 × MIC for 15 min at 37 °C and then quickly centrifuged (6000× *g*, 3 min). Subsequently, each sample was transferred to a black, opaque 96-well microtiter plate at 100 μL per well and divided into three separate wells. Then, a 100 μL aliquot of 2X staining solution (SYTO 9/propidium iodide (PI)) was added to each well, and the contents were stirred well. The dispersion was then incubated for 15 min at 25 °C in the dark, and the fluorescence of each bacterial suspension was assayed by confocal laser scanning microscopy (CLSM; Zeiss, Jena, Germany). The excitation/emission wavelengths were 485/535 nm for SYTO 9 and 485/610 nm for PI.

### 2.8. Field Emission Gun Scanning Electron Microscopy (FEGSEM) Analysis

Field emission gun scanning electron microscopy (FEGSEM) observation was performed based on a previously described protocol with slight modifications [24]. Cells of MDR *S. aureus* SA-596 and MDR *P. aeruginosa* PA-69 (approximately 10^8^ CFU/mL) were treated with verbascoside at a concentration of 0, MIC, or 2 × MIC and incubated at 37 °C for 4 h. After incubation, the cells were washed twice with PBS (pH 7.0) and then fixed with 2.5% glutaraldehyde for 12 h at 4 °C. The pellets were then washed twice with PBS prior to dehydration through an ethanol gradient (30%, 50%, 70%, 80%, 90%, and 100%). Following dehydration, the pellets were soaked in tert-butanol at −20 °C for 30 min. The lyophilized pellets were secured on a stage with conductive adhesive. Finally, the prepared samples were coated with a thin layer of gold and observed on an FEGSEM instrument (MLA 650, FEI, Hillsboro, OR, USA).

### 2.9. Biofilm Eradication Assays

Both tested bacterial strains were grown in TSB medium, and biofilm eradication assays were carried out as previously reported [25]. The strains were seeded in microplates (96-well plates) and incubated at 37 °C for 18 h. Following incubation, planktonic cells were removed. Subsequently, the remaining biofilm was stained with crystal violet and solubilized with 70% ethanol. Finally, the optical density at 562 nm was recorded as an indicator of the amount of biofilm eradication.

### 2.10. In Vitro Cytotoxicity

The cytotoxicity of verbascoside against various cell lines (HepG2, HEK 293 and A549 cell lines) was assessed using the CCK assay. Cells were seeded in 96-well plates (Corning, NY, USA) at a density of 8 × 10^3^ cells and incubated for 24 h in DMEM. Then, the cells were divided into ten groups: cells incubated with various concentrations (200.00 μM, 100.00 μM, 50.00 μM, 12.50 μM, 6.25 μM, 3.13 μM, 1.56 μM, 0.78 μM) of verbascoside solution, a blank group (without cells) and a negative control group (without verbascoside) for 24 h. Subsequently, the cell morphological changes were observed with an inverted microscope (Eclipse Ti-U, Nikon, Japan). After observing the cell morphology, 10 μL of CCK-8 solution was added to each well, and the absorbance at 450 nm was measured after 1 h of incubation using an enzyme marker (Thermo Fisher Scientific, Vantaa, Finland). The cell viability was calculated as follows: (OD of experimental group-OD of blank group)/(OD of negative control group-OD of blank group).

### 2.11. Total Number of Colony-Forming Units in Meat Samples

Fresh pork, beef, chicken and tuna purchased from local supermarkets were washed with sterile water after the fat and connective tissue were removed. The processed meat samples were sterilized under ultraviolet light for 1 h and then cut into evenly sized pieces of approximately 2 × 2 × 2 cm on an aseptic ultraclean worktable. An SA-596 bacterial suspension was added at a concentration of 10^8^ CFU/mL to the sample at a volume/mass ratio of 1:10, yielding a final concentration of 10^7^ CFU/g. Each type of meat was evenly divided into 4 groups, with 6 portions in each group. Then, 2 mL of verbascoside solution at a concentration of MIC, 2 × MIC, or 4 × MIC was evenly sprayed on the surface of each meat sample. In the control group, the verbascoside solution was replaced with the same amount of sterile water. This step was repeated three times for complete absorption. Subsequently, the meat samples were separately placed into sterile fresh-keeping bags and stored at 4 °C in the dark. The total number of colony-forming units in the meat samples was determined at 0, 1, 3, 5, 7, and 9 days of storage. One portion of the meat samples was cut into pieces and put into a conical flask filled with 90 mL of sterile normal saline and shaken for 3 min. One millilitre of the supernatant was collected, and 10-fold serial dilutions were prepared. Afterwards, 3 appropriate dilutions were selected, and the pour plate method was used to determine the total number of colonies after culturing at 37 °C for 2 days.

### 2.12. Statistical Analysis

The data were analysed using SPSS 25.0 software (IBM, Armonk, NY, USA). All of the assays were conducted in triplicate. The data are reported as the mean values ± standard deviations (SDs). Statistical analyses of the data were performed using one-way analysis of variance (ANOVA) followed by Tukey’s post hoc analysis at a 5% significance level [26].

## 3. Results

### 3.1. Minimal Inhibitory Concentration Values

Table 1 summarizes the results of the MIC tests. Six of the investigated bacterial strains exhibited ceftazidime resistance (MIC > 512 µg/mL), with lower MICs obtained for moxifloxacin (32 µg/mL) and levofloxacin (32–256 µg/mL). Moxifloxacin showed significantly higher antibacterial efficacy against all experimental MDR Gram-positive and Gram-negative bacteria. Furthermore, the MICs of verbascoside against the standard strains were greater than 1000 µg/mL, indicating a weak inhibitory effect. In contrast, verbascoside showed an obvious inhibitory effect with a low MIC value (625 µg/mL) against some MDR bacteria. Compared with the other isolates, SA-596 and PA-69 showed higher levels of antibiotic resistance, and hence, were employed for thorough mechanistic studies.

### 3.2. Evaluation of Synergistic Effects

The combined effects of verbascoside and antibiotics are shown in Table 2. Vancomycin is the preferred treatment option for severe staphylococcal infections. Ceftazidime is efficient against *P. aeruginosa* strains. Both of these antibiotics exhibit bactericidal effects by inhibiting the synthesis of bacterial cell walls. However, the efficacy of traditional cell wall synthesis-inhibiting antibiotics has been reduced by increasing bacterial resistance. In this experiment, the tested bacteria were insensitive to both of the commonly used antibiotics tested. Surprisingly, the FICI data suggested considerable synergistic effects of verbascoside in combination with antibiotics. The addition of verbascoside resulted in an eightfold reduction in the MIC of vancomycin against *S. aureus* ATCC 25923 (FICI = 0.375) and a sixteen-fold reduction in the MIC of vancomycin against MDR *S. aureus* SA-596 (FICI = 0.095). Similar MIC results were obtained with this combination against *P. aeruginosa*. When applied in combination with verbascoside, ceftazidime at 32 µg/mL was effective in inhibiting the growth of MDR *P. aeruginosa* PA-69 and reduced the MIC of ceftazidime by 32-fold (FICI = 0.0281). Thus, it can be concluded that a therapy combining verbascoside and widely used antibacterial drugs can restore or increase the activity of antibiotics against resistant pathogens.

### 3.3. Bacterial Growth Curve Analysis

**Figure 2:**
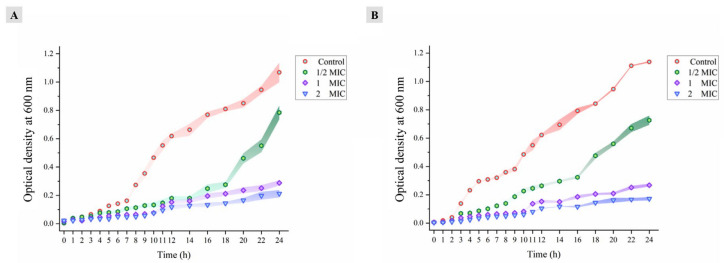
(**A**) MDR *Staphylococcus aureus* SA-596. (**B**) MDR *Pseudomonas aeruginosa* PA-69. The values are expressed as the mean ± standard deviation (n = 3).

The growth curves of the SA-596 and PA-69 strains in culture medium in the presence of different concentrations of verbascoside were plotted. As displayed in Figure 2, bacterial growth in the control group followed the model S-shaped curve, including the lag phase, logarithmic growth phase, and stationary phase. In the presence of verbascoside, MDR *S. aureus* SA-596 failed to enter the normal growth cycle (Figure 2A). Treatment with verbascoside at 1/2 × MIC led to a significant decrease in the growth rate of SA-596 but did not completely suppress growth; SA-596 entered the logarithmic growth phase after 14 h of incubation. After 8, 14 and 24 h of verbascoside treatment, the OD_600_ values were 41.48%, 27.10% and 73.51% of the control culture, respectively. This showed that bacterial growth was significantly slowed (*p* < 0.05). When SA-596 cells were treated with verbascoside at MIC or 2 × MIC, the bacteria did not enter the logarithmic growth phase, and there was no significant difference in OD_600_ between 8 and 24 h of incubation (*p* > 0.05), revealing a significant inhibitory effect on bacterial growth. Figure 2B shows the growth curve for *P. aeruginosa* PA-69 cells treated with various concentrations of verbascoside. Compared with the control treatment, a low verbascoside concentration (1/2 × MIC) delayed the growth of PA-69. However, treatment at the MIC and 2 × MIC significantly inhibited the growth of PA-69 in the logarithmic and stationary phases, and bacterial growth was almost completely inhibited by verbascoside at both treatment concentrations.

### 3.4. Membrane Permeability Assay

#### 3.4.1. Intracellular ATP Concentrations

**Figure 3:**
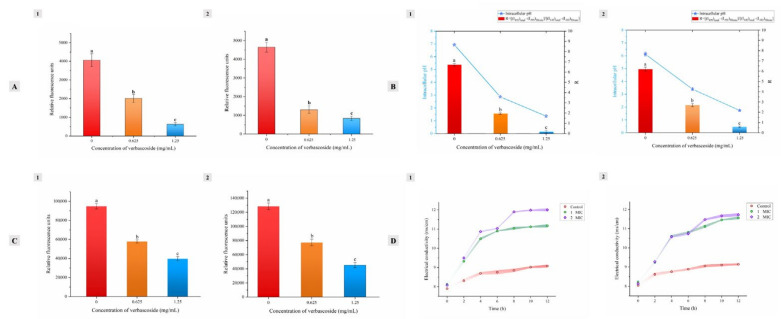
Effects of various concentrations of verbascoside on membrane permeability. (**A**) Intracellular ATP. (**B**) Intracellular pH. (**C**) Membrane potential. **(D**) Electrical conductivity. (1) MDR *Staphylococcus aureus* SA-596; (2) MDR *Pseudomonas aeruginosa* PA-69. The values are expressed as the mean ± standard deviation (n = 3). a–c Values with different lowercase letters are significantly different at *p* < 0.05.

ATP is the primary form of intracellular energy supply. Maintenance of the intracellular ATP level under bioenergetic stress is critical for cell survival. ATP cannot normally penetrate the intact plasma membrane of live cells, so leakage of ATP is one indicator of cell membrane injury [27]. The intracellular ATP concentration was estimated by a chemiluminescence assay. The ATP concentration and relative luminescence units indicated good linearity (y = 2.8442x + 1573.5, R^2^ = 0.9887). The results suggested that verbascoside caused a significant reduction (*p* < 0.05) in the intracellular ATP concentration compared with the control level in both strains (Figure 3A). In the control group without verbascoside, the fluorescence intensity from cellular ATP in SA-596 cells remained at approximately 4000. After verbascoside treatment at the MIC, the fluorescence intensity was reduced to approximately half this value (Figure 3A-1). Furthermore, the intracellular ATP level of the high-concentration verbascoside treatment group (2 × MIC) was reduced to approximately one-fifth that of the control group. The same concentration-dependent effect was found in the PA-69 strain (Figure 3A-2). Measurement of the intracellular ATP content indicated that treatment with verbascoside induced extensive ATP leakage from the cells. This leakage occurred because verbascoside forms channels in the cell membrane of bacteria that interrupt the proton motive force (PMF), causing a decrease in ATP concentration. Similarly, Yingjie Han et al. [28] reported that limonene reduced the cellular ATP level in *Listeria monocytogenes*, suggesting that a leakage of cellular components was induced by limonene.

#### 3.4.2. pH_in_

pH_in_ is crucially important in the control of several intracellular processes, such as protein synthesis, enzyme activity and DNA transcription. Cells with an intact membrane can maintain their pH_in_ via ion pumps and channels with changes in extracellular pH [29]. Therefore, in this study, pH_in_ was measured to confirm bacterial membrane damage by verbascoside. All assays were carried out in the presence of valinomycin to exclude the effects of other factors. The pH_in_ of normal SA-596 cells was 6.93 ± 0.11 (Figure 3B). After treatment with verbascoside, the cells exhibited a significant change in cytoplasmic pH. In the MIC treatment group, verbascoside caused a significant decrease (*p* < 0.01) in the SA-596 pH_in_ to 2.87 ± 0.08. Verbascoside at 2 × MIC led to a greater pH_in_ decrease to 1.36 ± 0.25 (*p* < 0.01). Similarly, verbascoside at the MIC and 2 × MIC significantly lowered the pH_in_ in PA-69 cells to 3.40 ± 0.12 and 1.74 ± 0.06, respectively, from 6.12 ± 0.16 Figure 3B-2. There was a significant concentration-dependent reduction in the pH_in_ as the concentration was changed from the MIC to 2 × MIC (*p* ≤ 0.01). Similarly, Vázquez-Sánchez et al. [30] reported that treatment with oregano essential oil, thymol, or carvacrol caused a reduction in the pH_in_ of *S. aureus* cells, demonstrating that membrane damage had occurred.

#### 3.4.3. Membrane Potential

The membrane potential was evaluated by determining the variation in DiBAC4 (3) fluorescence emission. DiBAC4 (3) enters depolarized cells and binds to proteins, leading to enhanced fluorescence [31]. In contrast, the fluorescence intensity is decreased in hyperpolarized membranes. SA-596 and PA-69 cells treated with verbascoside showed rapid membrane hyperpolarization, as demonstrated by a reduction in fluorescence (positive values) (Figure 3C). As the concentration of verbascoside increased from the MIC to 2 × MIC, the rate of decrease in the fluorescence intensity increased. Hyperpolarization is the most common type of membrane damage. Previous studies have shown that hyperpolarization is caused mainly by a change in pH or increased movement of ions, specifically K^+^, which diffuses out of the cell membrane through K^+^ channels and affects cellular homeostasis. Ultee et al. [32] reported that treatment with carvacrol caused a reduction in membrane potential and caused the membrane to become more permeable to protons, indicating the dissipation of ionic gradients as a result of pore formation.

#### 3.4.4. Electrical Conductivity

The effect of verbascoside on the conductivity of SA-596 and PA-69 suspensions is shown in Figure 3D. The conductivity of the normal bacterial suspension increased slowly with the extension of culture time. After the addition of various concentrations of verbascoside, the conductivity of the bacterial suspension increased very rapidly with increasing treatment time and did not stabilize until 8 h. When the strains were exposed to verbascoside at the MIC and 2 × MIC, the conductivity of the bacterial suspension increased in a concentration-dependent manner, reaching levels much greater than those in the control groups. After 12 h of treatment, compared with those of the corresponding control groups, the conductivities of the SA-596 (Figure 3D-1) and PA-69 (Figure 3D-2) suspensions treated with verbascoside at the MIC increased by 26.43% and 26.02%, respectively, and the SA-596 and PA-69 suspensions treated at 2 × MIC showed increases of 26.00% and 29.06%, respectively. These results indicated that verbascoside can enhance the permeability of the cell membrane in MDR *S. aureus* and *P. aeruginosa* and cause cellular electrolyte leakage. Similarly, Zhao et al. [33] suggested that sugarcane bagasse extract damaged the cell membranes of various bacterial species, as indicated by rapid increases in cytoplasmic conductivity.

### 3.5. Bacterial Membrane Integrity

**Figure 4:**
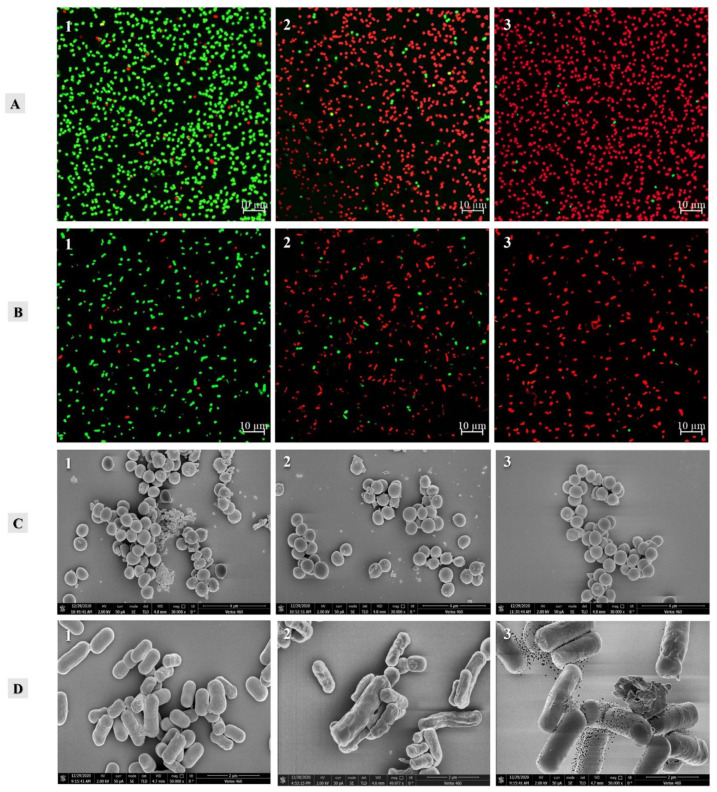
Effects of verbascoside on bacterial strains. (**A**) and (**B**) Cell membrane integrity of MDR *S. aureus* SA-596 and MDR *P. aeruginosa* PA-69. (**C**) and (**D**) Cell morphology of MDR *S. aureus* SA-596 and MDR *P. aeruginosa* PA-69. (1) Untreated bacterial strains. (2) Bacterial strains treated with verbascoside at the MIC. (3) Bacterial strains treated with verbascoside at 2 × MIC.

To further reveal the definite antibacterial mechanism of verbascoside, the bacterial membrane integrity was assayed. The effect of verbascoside on the integrity of the bacterial membrane was measured using CLSM after adding two fluorescent dyes. SYTO-9 is a cell-permeant dye that exhibits green fluorescence upon binding to the nucleic acids of both living and dead cells [34]. In contrast, PI enters only damaged cells and stains nucleic acids to produce red fluorescence [35]. Figure 4A-1,4B-1 show that the fluorescence of almost all the cells in the control group (untreated) was green, indicating that the cell membrane remained functionally intact. In contrast, in cells treated with verbascoside at the MIC, a significant decrease in green fluorescence and enhanced red fluorescence were observed. These results indicated that verbascoside induced damage to the cell membranes of SA-596 and PA-69 cells (Figure 4A-2,4B-2). After exposure to verbascoside at 2 × MIC, most of the cells were stained by PI (Figure 4A-3,4B-3), suggesting that the cell membrane permeability had been damaged by treatment with verbascoside.

### 3.6. FEGSEM Analysis

Morphological changes were investigated by FEGSEM. SA-596 cells treated with verbascoside did not show obvious membrane damage. The cells in the control group had normal coccal morphology with full and smooth surfaces (Figure 4C). However, previous cell membrane permeability experiments have confirmed that verbascoside can affect the cell membrane permeability of SA-596. The reason for this phenomenon may not be that verbascoside has a perforation effect on the cell membrane; rather, it may affect the voltage-gated ion channels on the cell membrane, resulting in increased cell membrane permeability. Notably, untreated SA-596 cells exhibited a large amount of biofilm formation. Such biofilms are highly resistant to host immune defences and are the main cause of bacterial resistance. The amount of biofilm was significantly reduced in the cells treated with verbascoside, and in the 2 × MIC treatment group, the biofilms were almost completely eliminated.

The effect of verbascoside on the morphology of PA-69 cells is shown in Figure 4D. When exposed to verbascoside at the MIC, cell morphological changes were evident, including slight wrinkling and folding of the cell surface (Figure 4D-2). When the concentration of the verbascoside treatment was 2 × MIC, clear alterations in cell morphology were observed, and there were extensive wrinkles and noticeable holes. In addition, extensive cell debris was scattered around the cells (Figure 4D-3). These results suggested that verbascoside caused morphological alterations in PA-69 cells. Similar morphological changes were observed in cells treated with bifidocin A in a previous study [36]. The results of that study suggested that the antibacterial effect of bifidocin A on *E. coli* might be due to pore formation on the cell membrane. In addition, in the present study, a small amount of biofilm formation was found among untreated PA-69 cells (Figure 4D-1). Therefore, biofilm eradication may be an important mechanism underlying the inhibitory effects of verbascoside on MDR bacteria.

### 3.7. Biofilm Eradication

**Figure 5:**
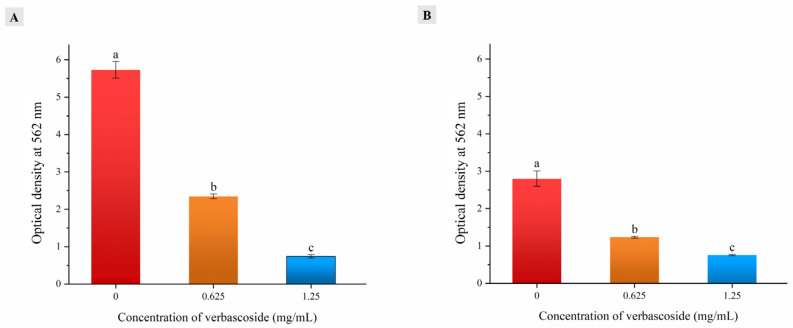
Effects of various concentrations of verbascoside on bacterial biofilms. (**A**) MDR *Staphylococcus aureus* SA-596. (**B**) MDR *Pseudomonas aeruginosa* PA-69. The values are expressed as the mean ± standard deviation (n = 3). a–c Values with different lowercase letters are significantly different at *p* < 0.05.

The biofilm eradication activity of verbascoside on MDR bacteria was assessed by the ability to eliminate preformed biofilms in a microtiter plate by a crystal violet staining assay. Under the assay conditions, both bacteria formed highly abundant biofilms, with 18 h preformed biofilms producing OD_562_ values of 5.73 and 2.80 (Figure 5). However, both bacteria showed significant biofilm reduction after verbascoside treatment. The OD_562_ values of SA-596 and PA-69 biofilms treated with verbascoside at the MIC decreased to 2.34 and 1.23, respectively. As the verbascoside treatment concentration increased, the OD_562_ values of the 2 × MIC treatment groups of SA-596 and PA-69 decreased by 87% and 73%, respectively, compared with the corresponding control values. Compared with those of the PA-69 cells, the biofilms of the SA-596 cells were more thoroughly eradicated by verbascoside, which is consistent with prior observations made by scanning electron microscopy. Poonacha et al. [37] reported a similar phenomenon in which an ectolysin named P128 eliminated coagulase-negative staphylococcal biofilms at low concentrations, thereby exerting an antibacterial effect.

### 3.8. In Vitro Cytotoxicity

**Figure 6:**
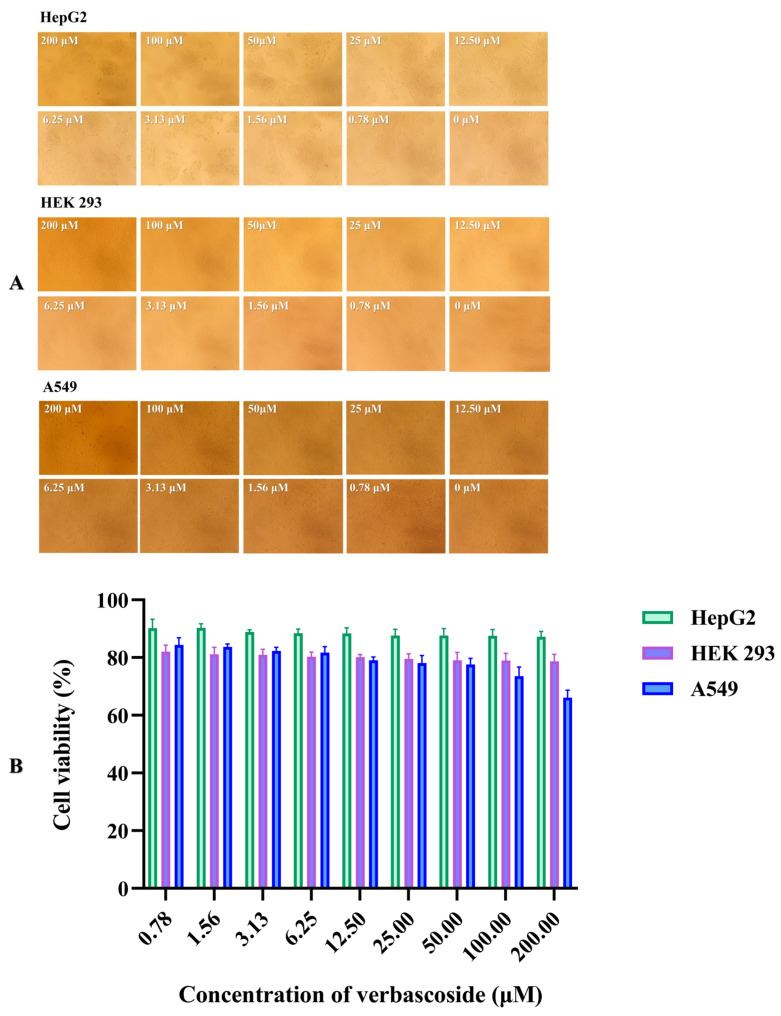
In vitro cytotoxicity of verbascoside. (**A**) Inverted microscopy images of various cells incubated with verbascoside at different concentrations; (**B**) cell viability of various cells treated with verbascoside.

It is important to develop natural products as surrogate antigens or additives with low toxicity or even no toxicity. By observing the morphological changes of the three cell lines treated with different concentrations of verbascoside, it was found that verbascoside at high doses (200 μM) did not change the cell morphology, nor did it affect the viability of these cells (Figure 6A). The edges of the cells were clear, and no cell fragments were observed. After the aforementioned observation, the cell viability was assessed by CCK-8 assays, as shown in Figure 6B. When the treatment concentration was 0.78 μM, the survival rates of HepG2, HEK 293 and A549 cells were 90.24%, 82.06% and 84.38%, respectively. Verbascoside treatment did not affect the survival rates of HepG2 and HEK 293 cells but did affect that of the A549 cell group; when the concentration was 200 μM, the survival rates were still 87.19% and 78.72%, respectively. This was similar to the outcomes under inverted microscopy. In addition, even at high concentrations, the survival rate of A549 cells was still 66.13%, indicating acceptable low toxicity.

### 3.9. Total Number of Colony-Forming Units in Fresh Meat

**Figure 7:**
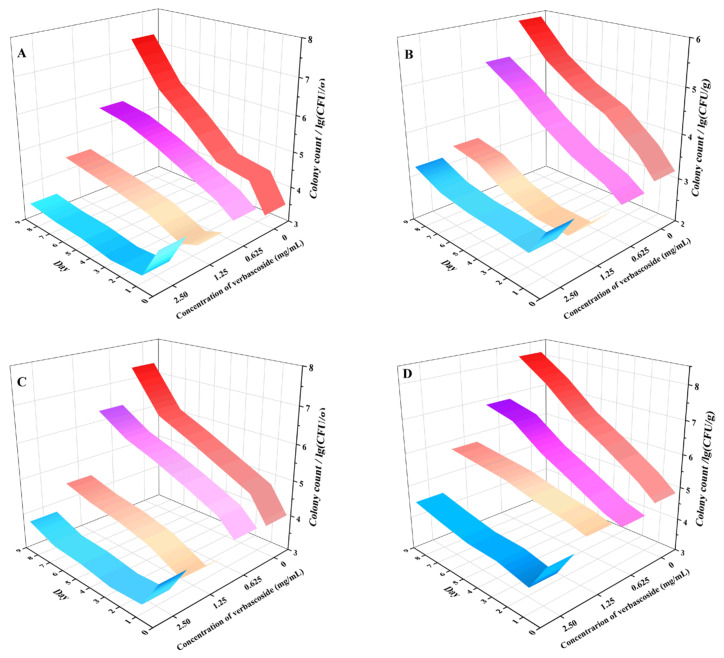
Changes in the number of colony-forming units in different meat samples during refrigeration with verbascoside treatment at various concentrations. (**A**) Chicken. (**B**) Beef. (**C**) Tuna. (**D**) Pork.

Figure 7 shows that in all the blank groups, the bacteria grew to the logarithmic phase when verbascoside was not administered. On the 7th day, the logarithm of the total number of colony-forming units in the beef group was greater than 5. In addition, the total number of colony-forming units in the other meat samples reached 10^6^, and the meat had deteriorated. Although the low dose (MIC) of verbascoside could slow the growth of SA-596 in all the meats, it failed to completely prevent the meat from spoiling. Compared with the blank group and the low-dose group, the medium-dose (2 × MIC) and high-dose (4 × MIC) groups showed a significant reduction in the total number of colony-forming units in the meat samples. Treatment with verbascoside at a concentration of 2 × MIC significantly inhibited the growth of spoilage bacteria in the meat samples and reduced the total microbial level in the meat samples during storage. By the end of the observation, when all the meat samples were refrigerated until the 9th day, the logarithm of the total number of colony-forming units in chicken, beef, tuna and pork was reduced by approximately half (*p* < 0.05). After treatment with verbascoside at a concentration of 4 × MIC, it was clearly observed that most of the bacteria in all the meat samples were dead. On the 1st day, the logarithm of the total number of CFUs in the chicken, beef, tuna and pork groups was significantly smaller than that on day 0, decreasing by 90%, 60%, 78% and 82%, respectively (*p* < 0.05). Furthermore, on the 9th day, the total number of colony-forming units in all the meat samples still did not increase significantly (*p* > 0.05). These results indicated that medium and high concentrations of verbascoside could significantly reduce the total number of colony-forming units in meat samples and prolong the shelf life of different types of meat samples.

## 4. Conclusions

In this study, verbascoside derived from edible natural plants was used as an adjuvant in combination with traditional cell wall synthesis-inhibiting antibiotics, and it was found that the addition of verbascoside strengthened the effects of the traditional antibiotics. Verbascoside exerts its antimicrobial activity through multiple mechanisms, including cell membrane dysfunction, biofilm eradication and changes in cell morphology. Therefore, the combination of verbascoside with traditional antibiotics had a significant antibacterial effect. Among all of the identified mechanisms, the alteration of bacterial cell membrane permeability is essential for the antibacterial activity of verbascoside. The cell membrane plays a vital role in maintaining optimal internal metabolism and energy transduction [38]. It has been suggested that compounds with more than one hydroxyl group predominantly target the cytoplasmic membrane through the accumulation of hydrophobic associates in the lipid bilayer. This process could interrupt lipid-protein communications; change the structure, function and permeability of the membrane; trigger the leakage of cellular contents; disrupt the PMF and electron influx; and ultimately disrupt intracellular homeostasis and destroy cell function [39]. Therefore, verbascoside has antimicrobial activity due to its unique multihydroxyl group chemical structure, which could perturb the lipid/water interface.

In addition, the highly hydrophilic character of verbascoside increases its range of possible applications. This characteristic confers certain advantages, such as good gastrointestinal absorption and adequate blood–brain barrier penetration [40]. Furthermore, Gram-positive bacteria are more sensitive than Gram-negative bacteria to antibiotics due to the presence of the outer membrane in the latter, which acts as a potential barrier to antimicrobial substances [41]. However, some porins (hydrophilic channels) in the outer membranes of Gram-negative bacteria are conducive to the entry of hydrophilic compounds [42]. Therefore, the high hydrophilicity of verbascoside is beneficial for its effects against Gram-negative bacteria.

When verbascoside was applied as an antibacterial food additive in fresh meat storage, it was found that although the medium and high doses of verbascoside could effectively kill bacteria in all the samples, the spoilage rates of different meats in the control and low-dose groups were slightly different. The biological components of different kinds of meat could differ, leading to differences in the degree of decay. Beef, chicken, and tuna all exhibit high protein, low fat, and low cholesterol levels. The protein content of beef and tuna is as high as 22% and 26%, respectively, while the fat content is only approximately 5% [43,44,45]. In contrast, pork is a fatty meat, with a fat content of approximately 25–30% and a protein content of less than 20%, exhibiting the highest fat content among meats [46]. It is speculated that among several fresh meats, pork has a relatively loose texture and extremely high fat content, which is one of the reasons for its high perishability. This will be a topic for further in-depth research.

In conclusion, treatment with verbascoside as a natural antibacterial agent maintained the freshness and prolonged the shelf life of a variety of fresh meats. Verbascoside may have potential as a novel agent for use in food preservation that can be applied to improve the safety and quality of fresh foods and curb bacterial contamination in many sectors, including the food, aquaculture and animal husbandry industries.

## Figures and Tables

**Table 1:** Antimicrobial activity expressed as the minimal inhibitory concentration.

Bacterial Strain		MIC (µg/mL)	MIC (µg/mL)		
		Verbascoside	Moxifloxacin	Levofloxacin	Ceftazidime
*S. aureus*	ATCC 25923	2500	32	256	>512
MDR *S. aureus*	SA-575	2500	32	32	>512
	SA-596	625	32	256	>512
*P. aeruginosa*	ATCC 15442	1250	32	256	>512
MDR *P. aeruginosa*	PA-69	625	32	256	>512
	PA-261	2500	32	128	>512

MIC: minimal inhibitory concentration; MDR: multidrug-resistant; *S. aureus*: *Staphylococcus aureus*; *P. aeruginosa*: *Pseudomonas aeruginosa*.

**Table 2:** Results of the application of verbascoside combined with antibiotics against *Staphylococcus aureus* and *Pseudomonas aeruginosa*.

Bacterial Strain		Agent	MIC (µg/mL)			
			Alone	In Combination	FICI	Outcome
*S. aureus*	ATCC 25923	Verbascoside	2500	625	0.375	Synergistic
		Vancomycin	1024	128		
MDR *S. aureus*	SA-575	Verbascoside	2500	2500	2.000	Neutral
		Vancomycin	512	512		
	SA-596	Verbascoside	625	20	0.095	Synergistic
		Vancomycin	1024	64		
*P. aeruginosa*	ATCC 15442	Verbascoside	1250	20	0.516	Partially synergistic
		Ceftazidime	1024	512		
MDR *P. aeruginosa*	PA-69	Verbascoside	625	156	0.281	Synergistic
		Ceftazidime	1024	32		
	PA-261	Verbascoside	2500	2500	1.125	Neutral
		Ceftazidime	1024	128		

MIC: minimal inhibitory concentration; MDR: multidrug-resistant; *S. aureus*: *Staphylococcus aureus*; *P. aeruginosa*: *Pseudomonas aeruginosa*.

## Data Availability

Not applicable.

## Sample Availability

Samples of the compounds are available from the authors.
